# The tandemly repeated NTPase (NTPDase) from *Neospora caninum* is a canonical dense granule protein whose RNA expression, protein secretion and phosphorylation coincides with the tachyzoite egress

**DOI:** 10.1186/s13071-016-1620-4

**Published:** 2016-06-21

**Authors:** Iván Pastor-Fernández, Javier Regidor-Cerrillo, Gema Álvarez-García, Virginia Marugán-Hernández, Paula García-Lunar, Andrew Hemphill, Luis M. Ortega-Mora

**Affiliations:** SALUVET, Animal Health Department, Faculty of Veterinary Sciences, Complutense University of Madrid, Ciudad Universitaria s/n, 28040 Madrid, Spain; Institute of Parasitology, Vetsuisse Faculty, University of Berne, Länggass-Strasse 122, CH-3012 Berne, Switzerland

**Keywords:** *Neospora caninum*, Dense granule protein, NTPase, Tachyzoite lytic cycle

## Abstract

**Background:**

NTPases (also NTPDases) are enzymes with apyrase activity. They are widely distributed among eukaryotes, and also among members of the family Sarcocystidae. In *Toxoplasma gondii,* the TgNTPase accumulates in the dense granules, and has been commonly associated with the strain virulence. In the closely related *Neospora caninum*, the NcNTPase lacks nucleoside diphosphate hydrolase activity and appears to be more abundant in virulent isolates, indicating that it may contribute to the pathogenicity of neosporosis. However, so far no additional information on NcNTPase has been provided.

**Methods:**

Herein, the Nc*NTPase* coding sequences were analysed by different *in silico* and *de novo* sequencing approaches. A comparative analysis of NcNTPase and NcGRA7 in terms of protein dynamics, secretion, phosphorylation, and mRNA expression profiles during the tachyzoite lytic cycle was also carried out. Moreover, NcNTPase immunolocalization was analysed by confocal microscopy techniques over a set number of time-points.

**Results:**

We describe the presence of three different *loci* containing three copies of the Nc*NTPase* within the Nc-Liv genome, and report the existence of up to four different Nc*NTPase* alleles in Nc-Liv. We also provide evidence for the occurrence of diverse protein species of the NcNTPase by two-dimensional gel electrophoresis. Both NcNTPase and NcGRA7 were similarly up-regulated and secreted during the egress and/or early invasion phases, and were phosphorylated. However, its secretion was not affected by the addition of calcium modulators such as A23187 and ethanol. NcNTPase and NcGRA7 localized in dense granules and parasitophorous vacuole membrane throughout the lytic cycle, although differed in their inmunolocalization during early invasion and egress.

**Conclusions:**

The present study reveals the complexity of the Nc*NTPase loci* in *N. caninum*. We hypothesize that the expression of different isoforms of the NcNTPase protein could contribute to parasite virulence. Our findings showed regulation of expression, secretion and phosphorylation of NcNTPase suggesting a potential role for progression through the tachyzoites lytic cycle.

**Electronic supplementary material:**

The online version of this article (doi:10.1186/s13071-016-1620-4) contains supplementary material, which is available to authorized users.

## Background

*Neospora caninum* is an apicomplexan cyst-forming parasite that causes abortion in cattle and neuromuscular disorders in canids. Rapidly replicating tachyzoites are responsible for parasite dissemination and harmful effects within the infected host, resulting in vertical transmission and abortion [1]. Host tissue damage occurs as a consequence of the tachyzoite lytic cycle, a tightly regulated process that enables parasite propagation with devastating effects for the infected cells [2, 3]. The lytic cycle has been extensively studied in the closely related parasite *Toxoplasma gondii* [4, 5], but only scarcerly investigated in *N. caninum*. However, preliminary studies showed that the molecular mechanisms that control this process are highly conserved among *T. gondii* and *N. caninum* [6].

Micronemes, rhoptries, and dense granules are secretory organelles exclusively found in apicomplexan parasites. The contents of these organelles are sequentially released during the lytic cycle, and play a crucial role in the host-parasite interactions. Specifically, dense granule proteins (GRA) are secreted into the parasitophorous vacuole (PV) and modify the PV membrane (PVM). The PV acts as a metabolically active compartment designed to favour parasite replication [7, 8]. More than 20 GRA proteins have been reported for *T. gondii* [9], and 15 have been identified in *N. caninum* at protein and transcriptional levels [10–12]. Nevertheless, only a limited number of GRA proteins have been studied in *N. caninum*, despite the fact that some GRA proteins such as TgGRA7, TgGRA15, TgGRA16, TgGRA24, TgGRA25 and TgNTPase I, among others, contribute to virulence in *T. gondii* [13–17].

The *N. caninum* GRA7 protein was extensively characterized during the last few years [10, 18, 19], whereas little information is available for the NcNTPase [20]. This protein appears to be more abundant in virulent isolates, suggesting that its function could be related with parasite virulence [21]. Besides, multiple genes coding for NTPase have been identified in both, *N. caninum* and *T. gondii* [20, 22]. In fact, *T. gondii* tachyzoites express two NTPase isoforms (NTPase 1 and 3, also termed NTPase II and I, respectively) which differ in their enzymatic activities, although TgNTPase 3 (with nucleoside triphosphate hydrolase activity) is restricted to the virulent type I strains [13, 23]. In previous studies, TgNTPase inhibition by antisense RNA compromised parasite replication, suggesting that NTPase activity is essential for parasite function [24]. Despite previous predictions, in a recent study deletion of the genes encoding either or both of the NTPase enzymes had no effect on growth or virulence in mice of *T. gondii* [25]. Only nucleoside triphosphate hydrolase activity has been found in tachyzoite extracts of *N. caninum* [20], and whether NcNTPase contributes to *N. caninum* virulence is still unknown.

We here have gone into detail about *NTPase* gene organization and present a comparative analysis of NcNTPase and NcGRA7 in terms of protein dynamics, secretion, phosphorylation, and mRNA expression profiles during the tachyzoite lytic cycle. This study increases the limited existing knowledge on these GRA proteins in *N. caninum*, and will serve as reference for future studies intended to establish their functional role during the proliferative phase of *N. caninum.*

## Methods

### *In silico* sequence analysis

All sequence data were obtained from ToxoDB v24 (www.toxodb.org), aligned using the CLUSTAL Omega and MUSCLE tools (www.ebi.ac.uk), and edited using the BioEdit software v7.1.1. BLAST tool from the NCBI website (www.ncbi.nlm.nih.gov/BLAST) was employed to match homologous sequences. Protein families were acquired from Pfam database (pfam.sanger.ac.uk). Promoter region sequences were also analysed with the Regulatory Sequence Analysis Tools (RSAT) for protists (rsat01.biologie.ens.fr/rsat/) [26].

### Parasite culture

The Nc-Liv isolate [27] was propagated in vitro by continuous passage in MARC-145 cell culture using standard procedures [28]. For transmission electron microscopy (TEM), murine epidermal keratinocyte cultures were infected with the Nc-Liv isolate as described earlier [29].

### Production of recombinant proteins and polyclonal antibodies

rNcNTPase and rNcGRA7 proteins were obtained as previously described [30]. Briefly, proteins were cloned in the pET45b(+) expression system (Novagen), expressed in *E. coli* BL21(DE3) pLysS competent cells (Agilent Technologies) as a (His)6-tagged fusion proteins, and purified using HisTrapHP columns coupled to the ÄKTAprime Plus system (GE Healthcare). All proteins were analysed by one-dimensional SDS-PAGE (1-DE) to check their purity and integrity. Electrophoresed proteins were manually excised from prepared Coomassie-stained gels for peptide mass fingerprinting (PMF) following standard procedures [31].

Polyclonal antibodies (PAbs) against rNcNTPase were raised in two female New Zealand White rabbits as previously described [32]. Polyclonal and monoclonal antibodies (MAbs) against rNcGRA7 were obtained as previously described [10, 19]. Affinity purified antibodies were prepared following standard procedures [19].

### One-dimensional and two-dimensional gel electrophoresis immunoblot

The NcNTPase protein was detected on Nc-Liv parasite extracts by 1-DE immunoblot as previously described [32]. PAbs α-rNcNTPase were used as primary antibody, whereas goat anti-rabbit IgG antibody conjugated to peroxidase (Sigma-Aldrich) was used as secondary antibody. Both antibodies were diluted at 1:1000. Reactions were developed using 4-chloro-1-naphtol (Bio-Rad) as substrate until signal visualization.

For two-dimensional gel electrophoresis (2-DE) immunoblot Nc-Liv tachyzoites were purified in desalting columns and protein extraction was performed as previously described [33]. Briefly, 2 × 10^8^ frozen tachyzoites were resuspended in 200 μl of lysis buffer (6 M urea [Sigma-Aldrich], 2 M thiourea [Fluka], 4 % CHAPS [Fluka], 65 mM DTE [Calbiochem], 10 mM Tris-HCl [Panreac] and 1 mM PMSF [Sigma-Aldrich]), processed by 3 cycles of freezing and thawing and solubilized by the addition of 200 μl of rehydratation buffer (8 M urea, 2 M thiourea, 2 % CHAPS, 65 mM DTE, and 1 % ampholyte [Bio-Rad]). Insoluble material was removed by centrifugation at 13,000 rpm for 30 min at 4 °C and protein concentration of the resulting supernatants were determined by the Bradford method (Bio-Rad) using bovine serum albumin (BSA) as standard. Protein extracts (100 μg) were resolved by 2-DE as previously described [33]. Isoelectric focusing (IEF) was performed on 17 cm-ReadyStrip™ IPG Strips pH 3–10 NL (Bio-Rad) with a Protean IEF cell system (Bio-Rad). Before the second-dimension separation, proteins on the strips were reduced with 4 % DTE and then alkylated with 5 % iodoacetamide in equilibration buffer (6 M urea, 50 mM Tris-HCl pH 6.5, 30 % glycerol, and 2 % SDS). Second-dimension electrophoresis and immunoblotting was performed as previously described [33]. After 2-DE, gels were transferred at 18 °C onto PVDF membranes for immunoblotting. The blotted membrane was blocked with TBS-Tween 20 buffer containing 5 % (w/v) dry milk and incubated with the PAb α-rNcNTPase at a 1:50,000 dilution. Then, the membranes were incubated with a goat anti-rabbit IgG antibody conjugated to peroxidase at a 1:100,000 dilution (Sigma-Aldrich). Blots were exposed for 1–30 s using the Immobilon Western Chemi-luminescent HRP Substrate (Millipore). AGFA CP1000 processor and AGFA films (Curix/RP2 Plus, 18 × 24 cm) were used for image acquisition. Image capturing was performed using the PDQuest™ (Bio-Rad) program.

### Transmission electron microscopy

TEM experiments were carried out as previously described [31]. Infected keratinocyte cultures were fixed, LR-White embedded and labeled with affinity-purified rabbit α-rNcNTPase at a dilution of 1:2, and goat anti-rabbit conjugated to 10 nm diameter gold particles diluted at 1:5, both in PBS/0.3 % BSA (Amersham). After extensive washing in PBS, the grids were air-dried, and then contrasted with uranyle acetate and lead citrate [2]. Specimens were viewed on a Phillips 600 TEM operating at 60 kV.

### Secretion assays

Secretion assays were performed as previously described [32]. Briefly, 1 × 10^8^ tachyzoites were suspended in 500 μl cold phenol red-free DMEM and stimulated with 10 μM A23187 (Sigma-Aldrich), 1 % ethanol (Merck Chemicals), or 10 mM dithiothreitol (DTT, Calbiochem) for 20 min at 37 °C. Non-stimulated parasites were suspended in 500 μl cold phenol red-free DMEM and kept on ice. Secretory fractions in the supernatants were recovered by centrifugation, filtered and supplemented with phosphatase and protease inhibitor cocktails (Sigma-Aldrich). Pelleted parasites were resuspended in cold PBS, supplemented with phosphatase and protease inhibitor cocktails, and recovered by centrifugation. All samples were stored at -80 °C until further analyses.

Pellets were resuspended in 1× Laemmli sample buffer, and supernatans were dissolved in 5× Laemmli sample buffer to the same final volume (600 μl). In order to estimate protein secretion, equal amounts of secretion supernatants and tachyzoite lysates (20 μl/lane ~ 3 × 10^6^ tachyzoites) were loaded onto 1-DE gels for immunoblot analyses. Detection of NcTUBα in secretion supernatants was employed as tachyzoite lysis indicator, whilst NcMIC2 detection was used as positive control of secretion [34]. PVDF membranes were incubated with α-rNcNTPase, α-rNcGRA7 and α-rNcMIC2 at a dilution of 1:5000, and with α-TUBα at a 1:10,000 dilution (MAb, Sigma-Aldrich). Goat anti-rabbit IgG and goat anti-mouse IgG antibody conjugated to peroxidase were employed as secondary antibodies at 1:25,000 and 1:80,000 dilution, respectively (Sigma-Aldrich). Reactions were developed by chemiluminiscence as described above.

### Protein dynamics throughout the lytic cycle by immunofluorescence

The localization of NcNTPase and NcGRA7 proteins during the lytic cycle of *N. caninum* tachyzoites from 1 to 56 h post-infection (hpi) was studied by immunofluorescence following previously described protocols [32]. Ice-cold methanol, 2 % paraformaldehyde in PBS, or 2 % paraformaldehyde-0.05 % glutaraldehyde in PBS, were used as fixatives for 10 to 30 min. Blocked and permeabilised coverslips were labeled with MAb α-NcSAG1 as a tachyzoite surface marker (1:250 dilution) [35], and subsequently with affinity purified PAbs against rNcNTPase and rNcGRA7 (1:8 dilution). Alexa Fluor 488-conjugated goat anti-mouse IgG and Alexa Fluor 594-conjugated goat anti-rabbit IgG (Molecular Probes) were employed as secondary antibodies at a 1:1000 dilution. Nuclei were stained with 4’,6-diamidino-2-phenylindole (DAPI) dye and coverslips were mounted on glass slides with ProLong® Gold antifade reagent (Molecular Probes) for the microscopic visualization. In addition, in order to co-localise NcGRA7 and NcNTPase proteins, some coverslips were incubated with MAb α-NcGRA7 (1:25 dilution) and affinity purified PAb α-NcNTPase (1:8 dilution), and then with the Alexa Fluor 594-conjugated goat anti-mouse IgG and Alexa Fluor 488-conjugated goat anti-rabbit IgG at 1:1000 dilution, respectively.

Single 1 μm slices of immunofluorescence stainings were captured with a Leica TCS-SPE confocal laser-scanning microscope (Leica Microsystems) in the Department of Biochemistry and Molecular Biology IV of the Complutense University (Madrid). Image processing was performed using the LAS AF (Leica Microsystems) and the ImageJ software (NCBI, https://imagej.nih.gov/ij/https://imagej.nih.gov/ij/ ).

### Evaluation of Nc*NTPase* and Nc*GRA7* mRNA expression levels

Messenger RNA (mRNA) expression levels during the tachyzoite lytic cycle were determined by real-time reverse transcription PCR following a previously described protocol [32]. Samples were obtained at four representative time points, representing either recent invasion, PV maturation, exponential growth of parasites or tachyzoite egress. For this purpose, MARC-145 cultures were infected with the Nc-Liv isolate and maintained during 6, 24, 48 and 56 h. The effect of induced egress on mRNA expression levels of NcGRA7 and NcNTPase was also studied by treating the cultures at 48 hpi with 10 mM DTT for 45 min. Total RNA was extracted using Maxwell® 16 LEV simplyRNA Purification Kit and RNA integrity was checked by electrophoresis on agarose gels. Reverse transcription was carried out with the master mix SuperScript® VILO™ cDNA Synthesis Kit (Invitrogen) and resulting cDNA was diluted 1:20 and analysed by real-time PCR using the Power SYBR® Green PCR Master Mix (Applied Biosystems) in the ABI 7300 Real Time PCR System (Applied Biosystems). Primers used for amplification of NcNTPase, NcGRA7 and the housekeeping genes NcTubulin alpha (TUBα) and NcSAG1 are shown in Table 1. Plasmids containing DNA targets for real-time PCR were employed as standard curves (pET45b(+)-GRA7, pET45b(+)-NTPase, pGEM-T-NcSAG1, and pGEM-T-NcTUBα) [32]. A seven-point duplicate standard curve based on 10-fold serial dilutions was included on each run. The -ΔCt values were calculated by subtracting the Ct value of the normalizer genes from the Ct value of each sample. Relative fold increases or decreases were assessed by the 2^–ΔΔCt^ method [36], using the mean expression values at 24 h post-infection as baseline. Raw RNA samples were included in each batch of amplifications to confirm the absence of *N. caninum* genomic DNA. Data analyses of mRNA expression levels were carried out by Kruskal-Wallis and Dunn’s tests using GraphPad Prism v.6.01 software.

**Table 1:** Primers used to amplify Nc*NTPase*, Nc*GRA7*, Nc*SAG1* and Nc*TUB*α sequences by real time-PCR

Protein	ToxoDB accession number	Primer sequences	Reference	Length	Introns ^*a*^	Slope ^*b*^	R^2 *b*^
NcNTPase	NCLIV_068400	Fw-ATTGACCCCGACAGTATTCG Rv-ACGCTTGGAATCAACAGACCT	This study	129 bp; Pos. 283–411	No	-3.54	0.998
NcGRA7	NCLIV_021640	Fw-GAACAGCATGAAGGGGACAT Rv-CACCATCTGTAATGGCATCG	This study	130 bp; Pos. 97–226	No	-3.61	0.997
NcSAG1	NCLIV_033230	Fw-CGGTGTCGCAATGTGCTCTT Rv-ACGGTCGTCCCAGAACAAAC	[70]	150 bp; Pos. 504–653	No	-3.24	0.997
NcTUBα	NCLIV_058890	Fw-GGTAACGCCTGCTGGGAG Rv-GCTCCAAATCCAAGAAGACGCA	[71]	166 bp; Pos. 49–214	Yes*	-3.24	0.994

^a^Primers for intron-containing sequences were designed using cDNA as template. * Forward primer for NcTUBα amplification annealed at intron splice junction to prevent amplification of genomic DNA

^b^Descriptive values of real time-PCR from standard curves for each pair of primers are shown

### Phosphorylation assays

Phosphorylation assays were performed using *N. caninum* infected MARC-145 cells at 56 hpi [32]. Cell cultures were resuspended in alkaline phosphatase-compatible buffer (100 mM sodium chloride [Panreac], 50 mM Tris-HCl [Panreac], 10 mM magnesium chloride [Merck Chemicals], 1 mM DTT [Calbiochem], 0.2 % Triton X-100 [Merck Chemicals] and protease inhibitor cocktail [Sigma-Aldrich], pH 7.9) or in phosphatase inhibitor buffer (50 mM HEPES [Sigma-Aldrich], 100 mM sodium fluoride [Sigma-Aldrich], 2 mM sodium orthovanadate [Sigma-Aldrich], 2 mM EDTA [Sigma-Aldrich], 1 mM DTT, 0.2 % Triton X-100 and protease inhibitor cocktail). Extracts were disrupted on ice by bath-sonication and vortexed during 45 min. Alkaline phosphatase treatment (20 U CIP/2 × 10^7^ tachyzoites, New England Biolabs) was only applied on extracts resuspended in alkaline phosphatase-compatible buffer for 90 min at 37 ° C. Resulting extracts were stored at -80 °C until further analysis.

Tachyzoite extracts were separated by 12.5 % 1-DE supplemented with 25 μM Phos-Tag (Wako Pure Chemicals Industries) and 50 μM manganese (II) chloride (Merck Chemicals). After electrophoresis, gels were washed once in 0.1 M EDTA in transfer buffer and transferred onto nitrocellulose membranes. Membranes were incubated with α-rNcNTPase and α-rNcGRA7 PAbs, and then incubated with goat anti-rabbit IgG antibody conjugated to peroxidase. Reactions were developed using 4-chloro-1-naphtol as substrate until signal visualization.

## Results

### Molecular characterization of the Nc*NTPase* sequence

**Fig. 1:**
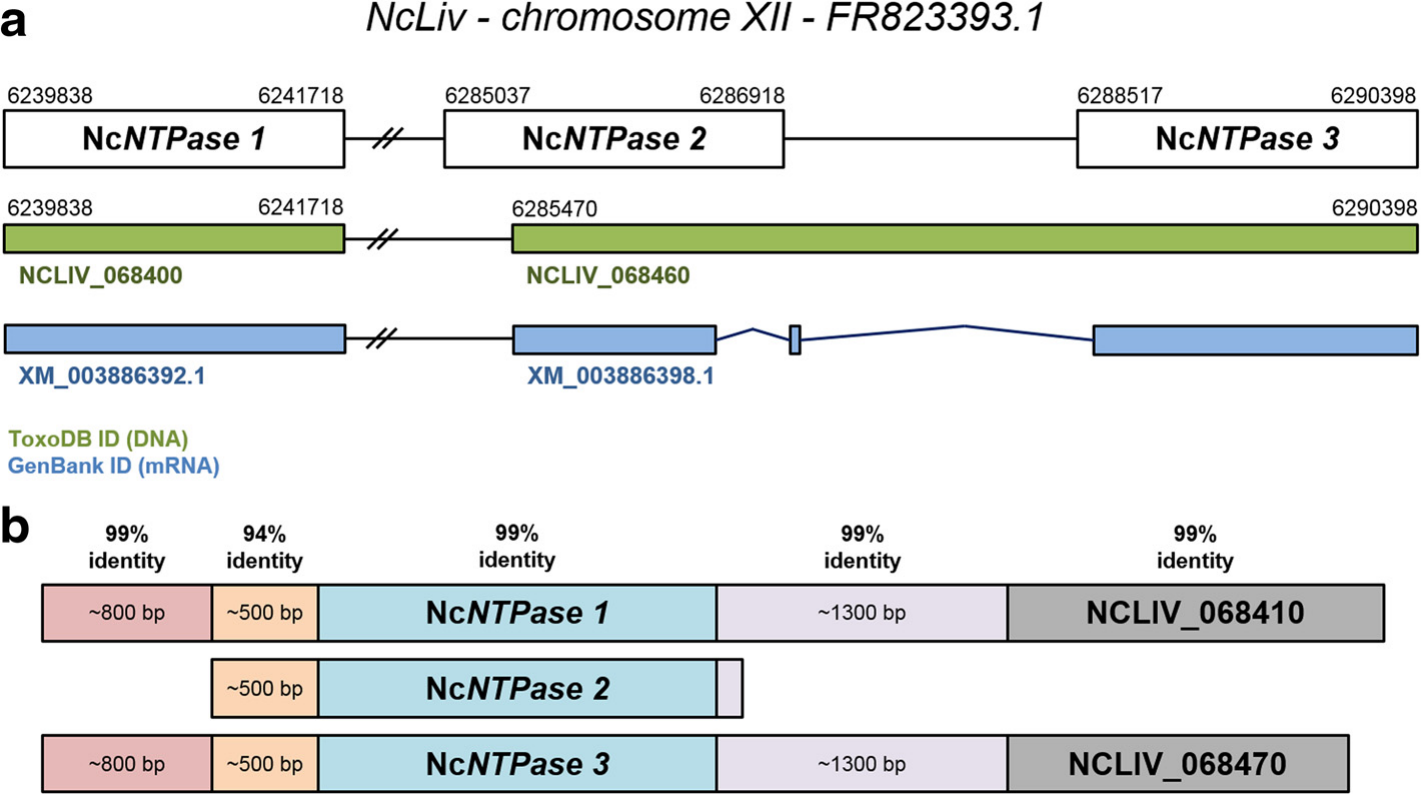
Diagram of the Nc*NTPase* structure in the Nc-Liv genome based on BLAST analyses. **a** Schematic alignment of the predicted *loci* (*first row*), the annotated genomic sequences (*second row*), and the annotated transcribed sequences (*third row*) of the Nc*NTPase* gene. Accession numbers, genomic positions and predicted introns are included on the graphic. **b** Schematic representation of the Nc*NTPase 1*, *2* and *3* gene structure as predicted in **a**. Identity scores between 5' and 3' flanking regions, and coding sequences are represented on the top of the figure

The Nc*NTPase* sequence is currently annotated under the gene ID NCLIV_068400 in the ToxoDB source (chromosome XII [GenBank: FR823393.1], position 6,239,838 to 6,241,718). It is classified as an unspecified product and considered as orthologous gene of Tg*NTPase 1* and *3*. The 1881 bp sequence has no introns and codes for a protein of a predicted molecular weight of ~68.7 kDa (Fig. 1a). The predicted protein sequence exhibits a signal peptide of 26 residues, and its EC number is 3.6.1.15 (nucleoside-triphosphate phosphatase). According to the Pfam database, the NcNTPase protein is a member of the GDA1/CD39 (nucleoside phosphatase) family.

Another gene displaying high similarity with the Nc*NTPase* sequence is NCLIV_068460, a predicted gene on chromosome XII, position 6,285,470 to 6,290,398. It codes for an unspecified product which displays 2 introns, with an overall length of 4929 bp and coding for predicted protein of ~109.2 kDa. BLAST analyses with NCLIV_068460 sequence showed that this sequence contains the C-terminus of a potential second Nc*NTPase* copy, and the whole nucleotide sequence of an additional copy. In order to clarify the Nc*NTPase loci* distribution, the Nc-Liv chromosome XII was analyzed in detail. In contrast to the information available in ToxoDB, three different *loci* containing three tandemly repeated copies of the Nc*NTPase* gene were predicted (Fig. 1a). These copies were termed Nc*NTPase 1* (position 6,239,838 to 6,241,718), Nc*NTPase 2* (6,285,037 to 6,286,918) and Nc*NTPase 3* (6,288,517 to 6,290,398) according to their spatial distribution within the genome (Fig. 1a). These analyses concluded that the NCLIV_068460 gene incorporate the last 1449 bp of Nc*NTPase 2* and 1553 bp of the full length of the Nc*NTPase 3*, and suggest that this gene has been mistakenly predicted (Fig. 1a).

In addition, BLAST searches for the Nc*NTPase* sequence NCLIV_068400 in the NCBI database produced significant alignments for four different mRNA annotations: two obtained from the Nc-Liv mRNAseq data [GeneBank: XM_003886392.1 and XM_003886398.1], and two obtained from the Nc-1 cDNA [GeneBank: AB525222.1 and AB010444.1]. The XM_003886392.1 sequence matched perfectly with the Nc*NTPase 1* allele (100 % identity), whereas the AB525222.1 and AB010444.1 annotations largely corresponded to the Nc*NTPase 2* and *3* alleles (~99 % identity), respectively (Additional file 1). The XM_003886398.1 annotation matched with the predicted mRNA sequence of the NCLIV_068460 gene.

Interestingly, the cDNA cloning experiments in this study resulted in the isolation of three additional Nc*NTPase* alleles from Nc-Liv, termed clone 1, 2, and 3 [GenBank: KU513388, KU513389, and KU513390, respectively] (Additional file 1). Hence, at least four different Nc*NTPase* alleles are transcribed in the Nc-Liv (XM_003886392.1, clone 1, clone 2, and clone 3). All new sequences display a high percentage of identity (>99 %) with the NCLIV_068400 gene (Nc*NTPase 1*), but carry a number of nucleotide changes that match with some of those observed in the Nc*NTPase 1*, *2*, and *3* copies (Additional file 1). Clone 1 displayed the highest identity with the Nc*NTPase 2* and *3* (with 5 nucleotide changes) and clones 2 and 3 with the Nc*NTPase2* (with 1 and 2 nucleotide changes, respectively) (Additional file 1). In this study, clone 3 was expressed in *E. coli* and the resulting recombinant protein was used to raise polyclonal antibodies in rabbits.

In order to gain more information on the NcNTPase *loci* distribution within the Nc-Liv genome, the flanking regions of the Nc*NTPase 1, 2* and *3* copies were also analysed in detail by BLAST. As shown in Fig. 1b, Nc*NTPase 1* and *3* share almost perfectly identical up- and down-stream sequences. In contrast, these elements were absent in the Nc*NTPase 2* gene. In order to confirm these findings, specific primers were designed to amplify the up-stream sequences of the Nc*NTPase 1*, *2* and *3*. Subsequently, the amplicons were sequenced and analyzed in detail (Additional file 2). Fragments comprising 1331, 536 and 1285 bp were obtained in two directions from the Nc*NTPase 1*, *2* and *3* up-stream regions, respectively. All the sequences were identical to the originally published ones and confirmed the upper distribution of the Nc*NTPase 1*, *2* and *3* copies (data not shown). RSAT analyses on upstream regions confirmed the presence of *cis*-regulatory motifs (TGAGACGC) within the up-stream regions. Specifically, three of these elements were found for the Nc*NTPase 1* and *2* copies, and four were found for the Nc*NTPase 3* (Additional file 2).

According to the Nc-Liv chromosome XII sequence, the Nc*NTPase 2* and *3* copies contain an additional nucleotide (1882 instead of 1881). This is due to the insertion of an additional G, at the position number 8, in a region with tandemly repeated Gs. Strikingly, this insertion alters the reading frame for the mRNA transcription. However, we confirmed the presence of this additional nucleotide by sequencing analyses of the up-stream region on genomic DNA (Additional file 2).

### Characterization of NcNTPase protein

The identity of the recombinant NcNTPase - clone 3 expressed in *E. coli* was corroborated by mass spectrometry (theoretical molecular weight: 69,9 kDa, Score: 214; mass values matched: 25/65; sequence coverage: 47 %). Hence, the recombinant protein was employed to develop specific polyclonal antibodies (PAbs).

**Fig. 2:**
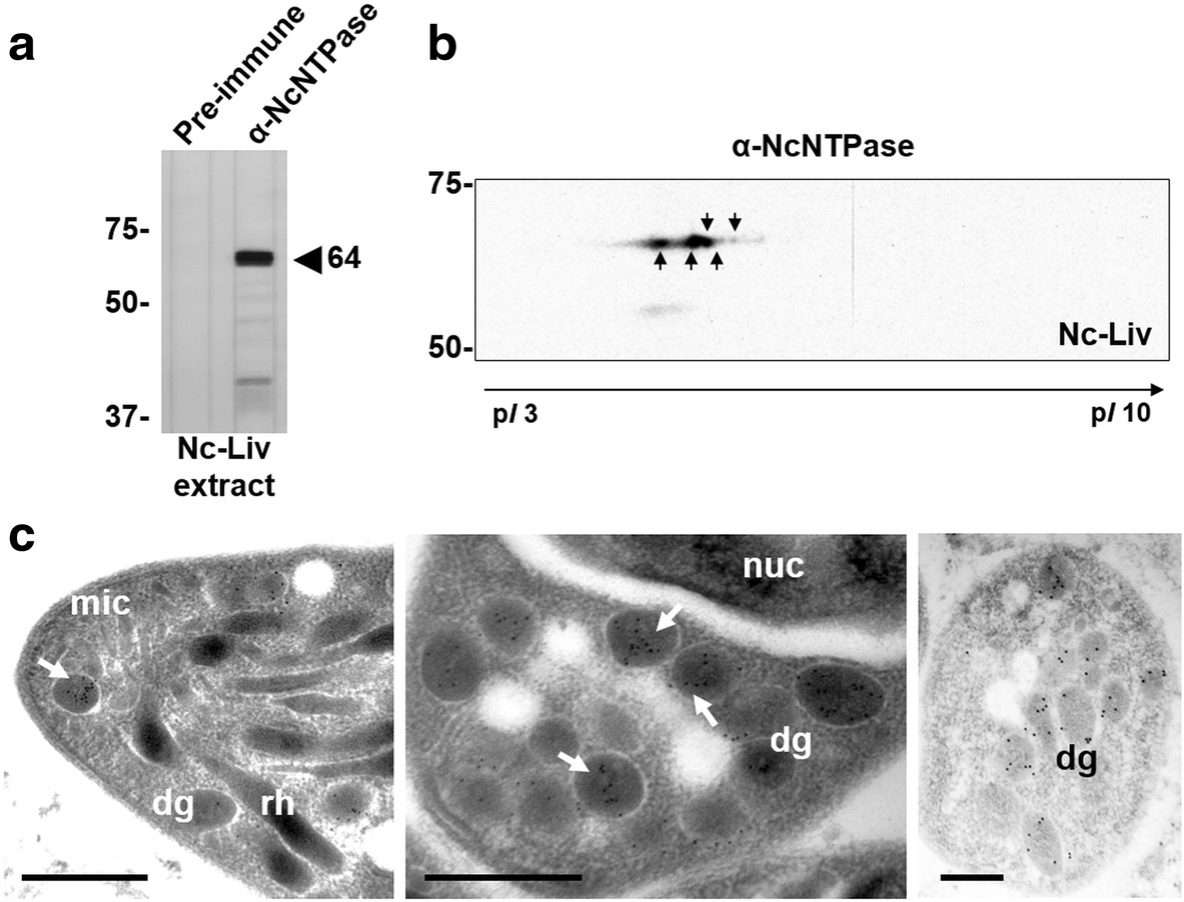
**a** *N. caninum*-based immunoblot showing the immuno-reactivity of α-rNcNTPase antibodies against parasite extracts. A main band of approximately 64 kDa was detected. **b** NcNTPase-immunome profile of the tachyzoite stage of the Nc-Liv isolate. Proteins were separated along a non-linear pH gradient (pH 3–10 NL, IPG strips; 17 cm) in the first dimension and on a 10 % polyacrylamide gel in the second dimension. Following transfer to PVDF membranes, a polyclonal rabbit antiserum raised against rNcNTPase was used. The 2-DE immunoblot was analyzed with the PD-Quest software. Arrows indicate the detected protein spots. **c** Immunogold TEM of *N. caninum* tachyzoites labelled with the α-rNcNTPase antibody. The images show in detail the apical region and the dense granules in longitudinal and cross sections. The NcNTPase is clearly associated with dense granules (*arrow*). Dense granules (dg), micronemes (mic), rhoptries (rh) and the nucleus (nuc) are indicated on the micrographs. *Scale-bars*: 0.5 μm

Immunoblotting of Nc-Liv extracts separated by 1-DE under reducing conditions revealed that the polyclonal α-rNcNTPase antiserum reacted with a main band of approximately 64 kDa (Fig. 2a). On immunoblots of Nc-Liv extracts separated by 2-DE, the polyclonal α-rNcNTPase antiserum recognized a chain of at least 5 spots located in the acidic range of the pH gradient with an approximate *Mr* of 60–70 kDa but different isoelectric points (protein species) (Fig. 2b). These findings suggest either the presence of multiple isoforms of the NcNTPase, or the occurrence of protein modifications such as phosphorylation. The same affinity purified antiserum was employed for immunogold TEM, which showed that the NcNTPase protein is clearly associated with the tachyzoites dense granules (Fig. 2c).

### Comparative analysis of Nc*GRA7* and Nc*NTPase* mRNA expression levels during the lytic cycle

**Fig. 3:**
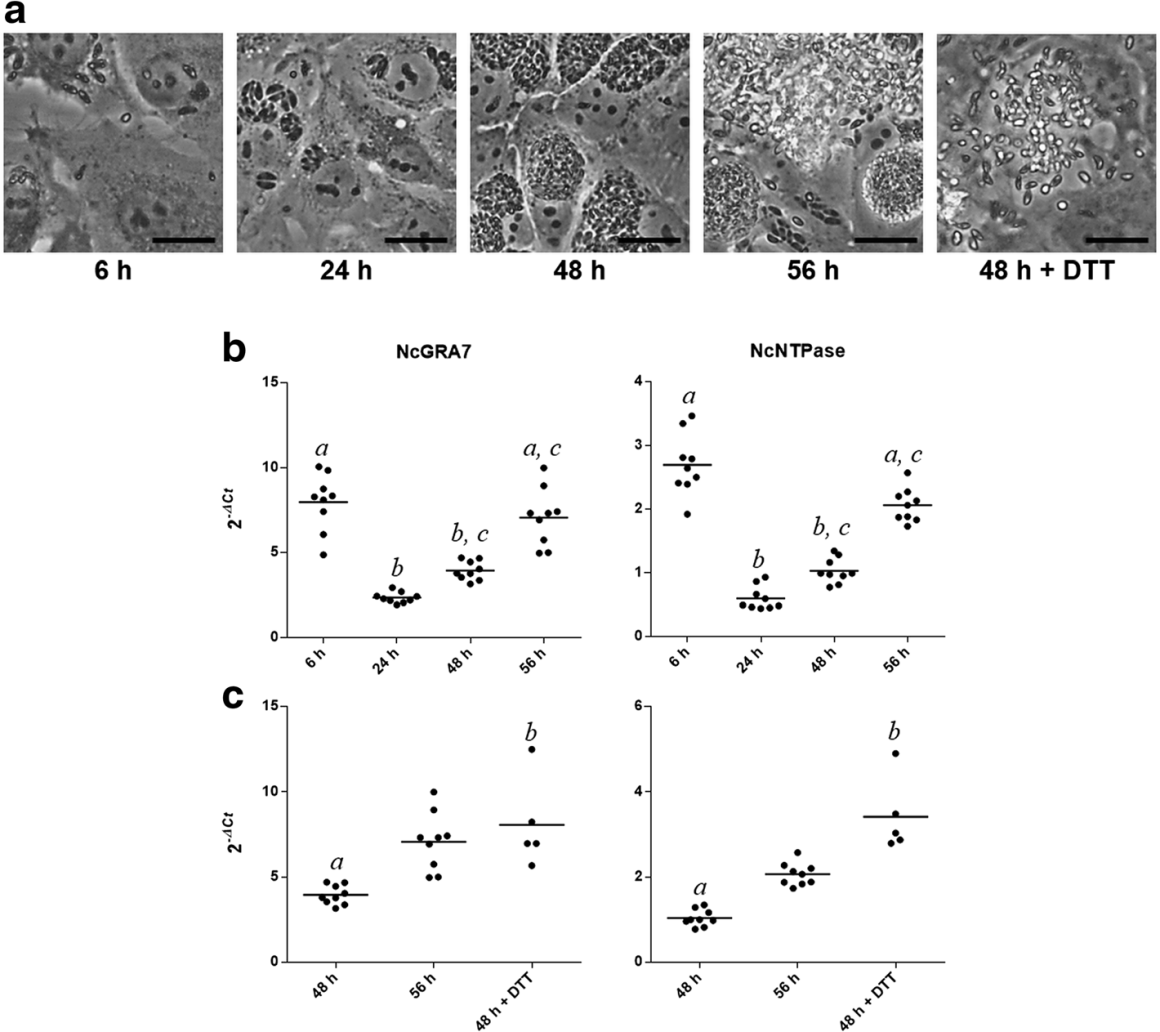
mRNA levels of NcGRA7 and NcNTPase. Real time-PCR was employed to quantify the mRNA transcription of both proteins along the lytic cycle. **a** Photomicrographs showing the infection dynamics of the Nc-Liv isolate on MARC-145 cultures at recent invasion (6 hpi), PV maturation (24 hpi), exponential growth of parasites (48 hpi) and tachyzoite egress (56 hpi and 48 hpi + DTT). *Scale-bars*: 20 μm. **b** mRNA levels of NcGRA7 and NcNTPase during the lytic cycle. **c** Effect of DTT supplementation to artificially induce egress at 48 hpi on mRNA levels for both proteins. For **a** and **b**, each point represents a single sample and bars represent the mean value. *a*, *b* and *c* indicate significant differences (Kruskal-Wallis test, followed by Dunn’s test)

Nc*NTPase* and Nc*GRA7* transcripts were quantified at different time-points, namely shortly after invasion (6 hpi), PV maturation (24 hpi), exponential growth of parasites (48 hpi) and tachyzoite egress (56 hpi), using Nc*TUBα* as normalizer gene. Both dense granule genes displayed a similar mRNA transcription pattern during the lytic cycle (Fig. 3a), showing the lowest mRNA levels at 24 hpi and the highest at 6 and 56 hpi [Kruskal-Wallis test, *H* = 29.95, *P* < 0.0001 for Nc*NTPase* and *H*  = 31.06, *P* < 0.0001 for Nc*GRA7,* followed by Dunn’s test*, P* < 0.0001 (6 hpi *vs* 24 hpi) and *P* = 0.0012 (56 hpi *vs* 24 hpi) for Nc*NTPase* and *P* < 0.0001 (6 hpi *vs* 24 hpi) and *P* = 0.0002 (56 hpi *vs* 24 hpi) for Nc*GRA7*]. Significant differences in mRNA levels were also observed at 48 hpi for both proteins [Dunn’s test, *P* = 0.0025 (6 hpi *vs* 48 hpi) for Nc*NTPase* and *P* = 0.0147 (6 hpi *vs* 48 hpi) for Nc*GRA7*]. Consequently, mRNA levels at 24 hpi were used as baseline to calculate transcript fold increases. Nc*GRA7* displayed a 3-fold increase in mRNA levels at 6 and 56 hpi. Similarly, Nc*NTPase* showed a 5-fold and a 4-fold increase at 6 hpi and 56 hpi, respectively. Both genes displayed a 2-fold increase at 48 hpi. Results were similar when Nc*SAG1* was used as a normalizer gene (data not shown).

Nc*NTPase* and Nc*GRA7* mRNA levels were also assessed after DTT stimulation, which leads to tachyzoite egress. Upon DTT treatment, tachyzoites exhibited significant increases in mRNA levels for both genes [Kruskal-Wallis test, *H* = 19.18, *P* < 0.0001 for Nc*NTPase* and *H* = 15.85, *P* < 0.0001 for Nc*GRA7*, followed by Dunn’s test, *P* < 0.0001 (48 hpi *vs* 48 hpi + DTT) for Nc*NTPase* and *P* = 0.0038 (48 hpi *vs* 48 hpi + DTT) for Nc*GRA7*] (Fig. 3b). Similarly to naturally occurring egress, Nc*GRA7* mRNA levels were 3-fold increased, while those coding for Nc*NTPase* displayed a 6-fold increase.

### Immunolocalization dynamics of NcGRA7 and NcNTPase throughout the lytic cycle

Herein, three different fixatives were employed in order to obtain a concise picture of the localization of these two dense granule proteins throughout the lytic cycle. In general, methanol fixation retained more efficiently the reservoirs of intracellular NcNTPase and NcGRA7, whilst paraformaldehyde and glutaraldehyde fixation further preserved the protein after secretion.

**Fig. 4:**
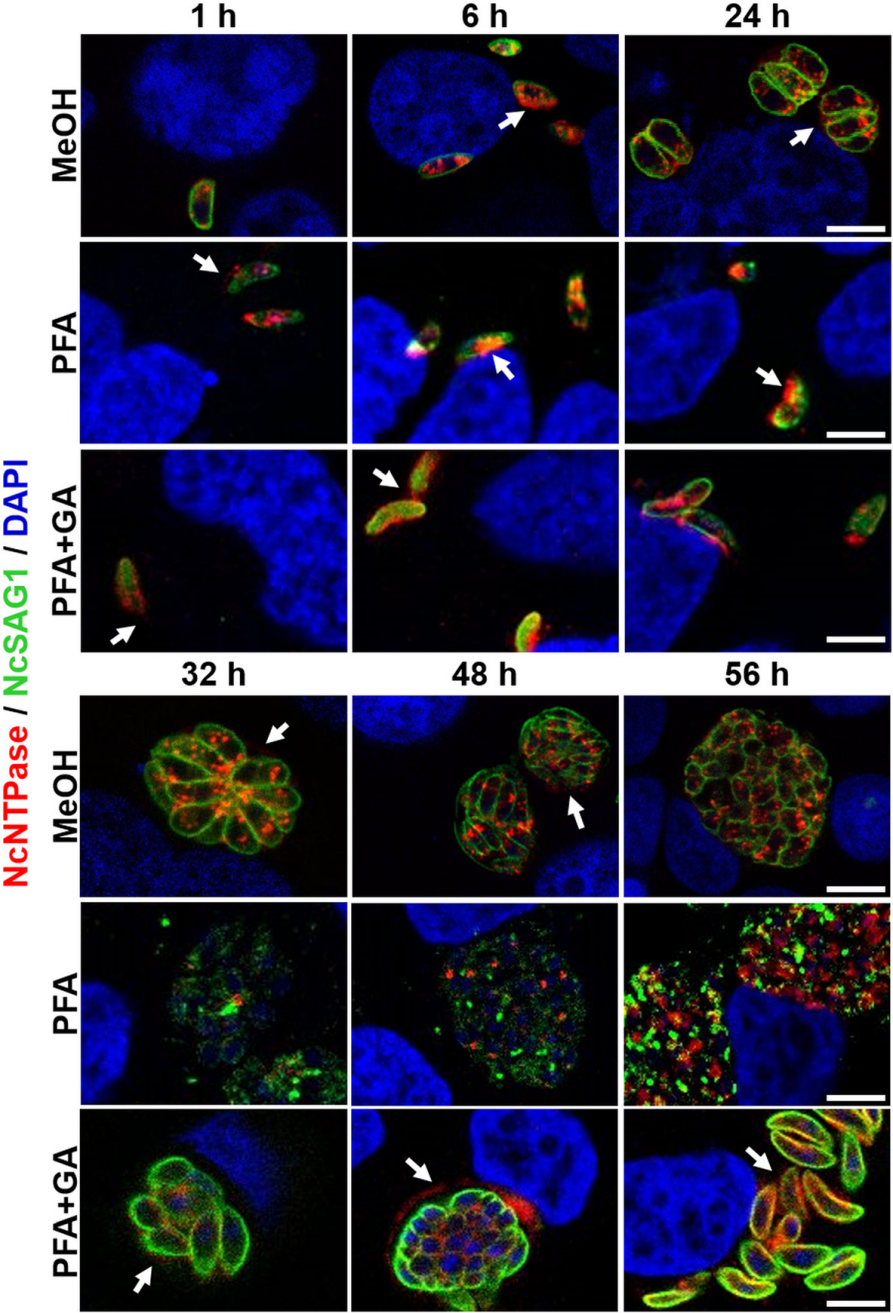
Confocal laser scanning microscopy of NcNTPase along the lytic cycle of tachyzoites. Infected cultures were fixed with methanol (MeOH), paraformaldehyde (PFA) and paraformaldehyde combined with glutaraldehyde (PFA + GA). Then, coverslips were double labelled with affinity purified antibodies against NcNTPase (*red*) and monoclonal antibodies against NcSAG1 (*green*). Nuclei were stained with DAPI (*blue*). All the images show a single 1 μm slice. *Scale-bars*: 4 μm

**Fig. 5:**
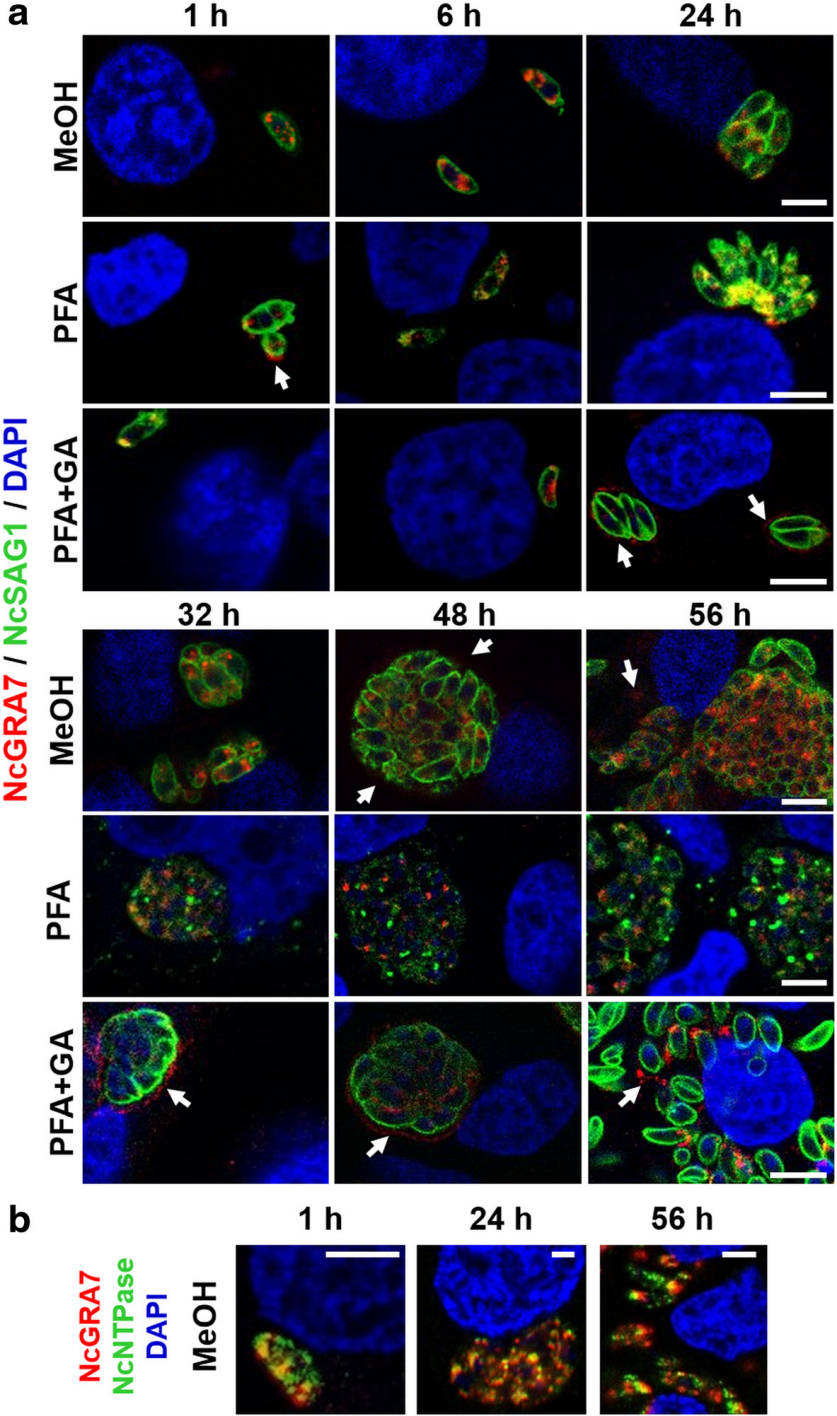
**a** Confocal laser scanning microscopy of NcGRA7 along the lytic cycle of tachyzoites. Infected cultures were fixed with methanol (MeOH), paraformaldehyde (PFA) and paraformaldehyde combined with glutaraldehyde (PFA + GA). Then, coverslips were double labelled with affinity purified antibodies against NcGRA7 (*red*) and monoclonal antibodies against NcSAG1 (*green*). Nuclei were stained with DAPI (*blue*). **b** Confocal laser scanning microscopy of NcNTPase (*green*) and NcGRA7 (*red*) along the lytic cycle of tachyzoites. Methanol-fixed cultures were double labelled with affinity purified antibodies against NcNTPase (*green*) and monoclonal antibodies against NcGRA7 (*red*). Images taken at 1 hpi, 24 hpi and 56 hpi show a single tachyzoite, a parasitophorous vacuole and a large group of tachyzoites undergoing egress, respectively. Nuclei were also stained with DAPI (*blue*). All the images show a single 1 μm slice. *Scale-bars*: 4 μm

Immunofluorescence staining using α-NcNTPase antibodies confirmed that NcNTPase is distributed throughout the tachyzoites cytoplasm exhibiting a punctate staining pattern that is reminiscent for dense granules (Fig. 4). NcNTPase secretion was clearly observed in most of the analyzed time points, and could be detected during early invasion, PV maturation, and egress. In addition, the NcNTPase localized to the PVM as soon as the PV was formed. This was particularly visible in samples fixed in the presence of paraformaldehyde and glutaraldehyde (Fig. 4). Compared to NcNTPase, the recognition pattern of α-NcGRA7 antibodies observed by immunofluorescence was less distinctive. Protein secretion was also evident during the early invasion, PV maturation and egress, and PVM association was observed (Fig. 5a).

On the other hand, co-immunolocalization studies revealed differences in the distribution pattern of the NcNTPase and NcGRA7 proteins during invasion (1 h) and egress (56 h). At these time points, NcNTPase was scattered throughout the entire tachyzoite cytoplasm, whereas NcGRA7 labeling was restricted to more specific areas close to the parasite surface. By contrast, during PV maturation (24 h), NcNTPase and NcGRA7 colocalized much more closely (Fig. 5b).

### Effects of calcium on NcNTPase and NcGRA7 secretion

**Fig. 6:**
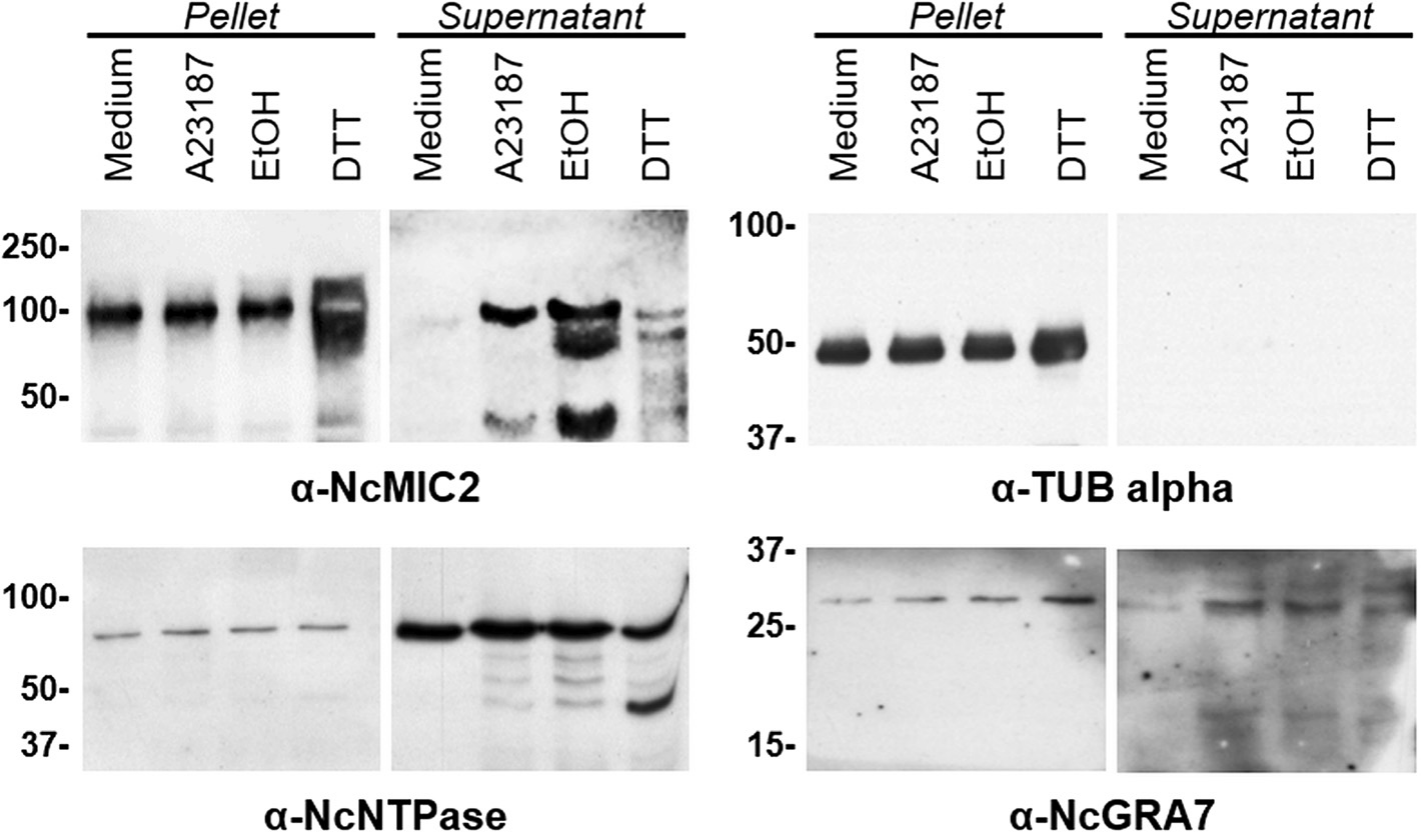
Effect of culture media (4 °C), A23187 (37 °C), ethanol (37 °C), and DTT (37 °C) on secretion of NcNTPase and NcGRA7 proteins as shown by Western-blot using respective antibodies. The same protein samples were also probed by immunoblot analyses with α-NcMIC2 and α-TUBα antibodies to confirm induced secretion and exclude inadvertent tachyzoite lysis, respectively. NcNTPase and NcGRA7 discharge was observed in culture supernatants regardless of the treatment. In contrast, NcMIC2 secretion was only evident after A23187, ethanol and DTT supplementation. Tachyzoite lysis was not detected. All the antibodies specifically reacted against their respective protein on tachyzoite extracts

Freshly purified tachyzoites were incubated in culture medium at 4 ° C, and in the presence of A23187, ethanol, and DTT (an egress inducer that activates NcNTPase in vitro) at 37 °C. Immunoblot analyses revealed that NcNTPase and NcGRA7 release was not apparently influenced by any chemical treatment. Both proteins were present in all secreted fractions of both treated and non-treated parasites (Fig. 6). Calcium-mediated protein secretion was validated by NcMIC2 secretion upon A23187, ethanol or DTT treatment, and tachyzoite lysis was discarded by the lack of detection of NcTUBα in all culture supernatant fractions (Fig. 6).

### Phosphorylation of NcNTPase and NcGRA7

**Fig. 7:**
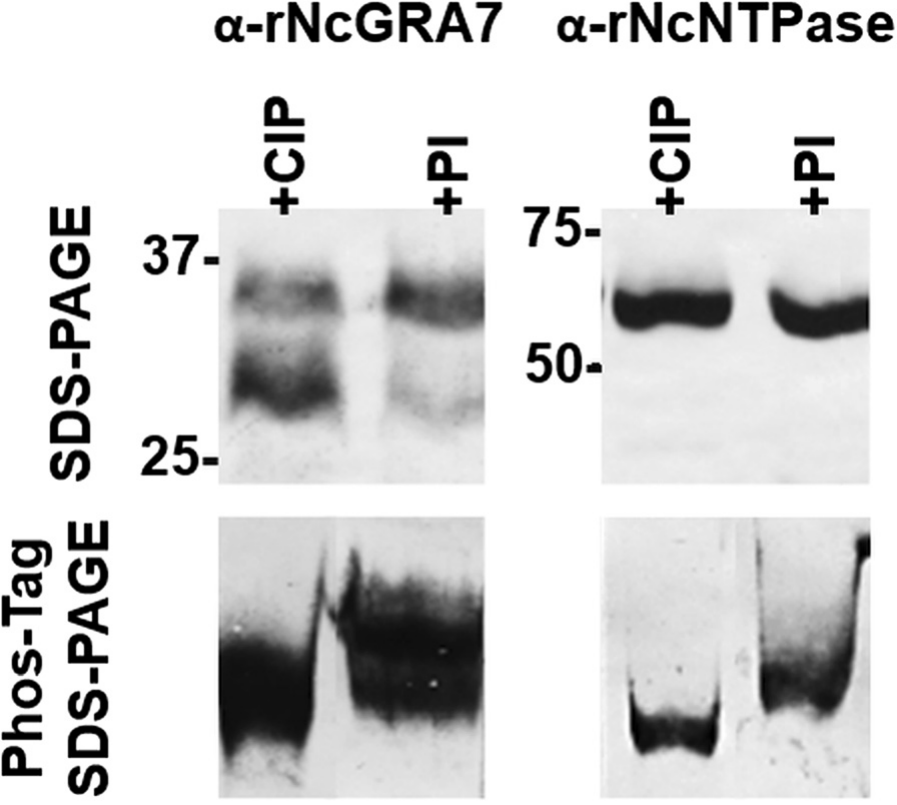
Phosphorylation detection of NcGRA7 and NcNTPase by Phos-Tag 1-DE. Tachyzoite extracts obtained at 56 hpi were processed with alkaline phosphatase (CIP) and phosphatase inhibitors (PI), electrophoresed on 1-DE and Phos-Tag 1-DE gels, and blotted to nitrocellulose membranes. Then, both proteins were detected by their respective antibodies in order to detect a mobility shift of the proteins treated with PI

Phosphorylation of NcNTPase and NcGRA7 was studied at 56 hpi to determine whether these proteins are likely to experience this post-translational modification. For this purpose, tachyzoite extracts were processed under conditions that preserve the phosphorylation status of each protein and resolved by Phos-Tag 1-DE. Both NcNTPase and NcGRA7 showed an electrophoretic mobility shift in those extracts treated with phosphatase inhibitors, which suggests that both proteins are phosphorylated at 56 hpi (Fig. 7) This is in contrast with our previous findings, in which the NcROP40 protein was not phosphorylated under the same conditions [32].

## Discussion

In the present work we have performed, for the first time, a comprehensive study on the localization dynamics, secretion, phosphorylation, and mRNA expression profiles of the two GRA proteins NcNTPase and NcGRA7 throughout the tachyzoites lytic cycle. Although the previously characterized NcGRA7 was included in this study mainly for comparative purposes, we present some new and undescribed aspects of this GRA protein. Moreover, we propose a new model for the Nc*NTPase* genomic distribution supported by different approaches.

In *N. caninum* the NcNTPase protein has been shown to be more abundant in virulent isolates [21]. However, several functional aspects of this protein still need to be elucidated. Overall, NTPases (also named as NTPDases in the literature) are found in almost all eukaryotes. These enzymes present apyrase conserved regions to hydrolyze nucleoside triphosphates and diphosphates in the presence of divalent cations [37]. The NTPases are also present in both *T. gondii* and *Sarcocystis neurona* [13, 20, 38]. Intriguingly, orthologues of *NTPase* are not found in *Eimeria* and *Plasmodium* parasites, indicating that this protein may participate in some conserved processes among the Sarcocystidae family members [20, 38]. In *T. gondii,* some NTPase isoforms are restricted to the virulent strains [13]. Nevertheless, there is no consensus regarding their specific function, although it has been proposed they participate in purine salvage to supply energy [39], to be involved in tachyzoite intracellular proliferation and egress [24, 40], and in the suppression of the host immune responses by interfering in purinergic signaling [37, 41–43]. Previous studies suggested that TgNTPase I was refractory to deletion, but in a new study each of the *NTPase* genes were easily deleted, as well as the double mutant was generated, showing that NTPase I and II were not essential for tachyzoite growth in vitro or virulence in mice. Nevertheless these findings do not rule out another role in different life cycle stages or different hosts [25], and also, the potential relevance of NTPase activity in *N. caninum*.

Herein, we describe the presence of three different *loci* for the Nc*NTPase* gene within the Nc-Liv genome. Our sequence analyses showed that Nc*NTPase 2* lacks the up and down-stream elements shared by Nc*NTPase 1* and *3*. Similarly, *T. gondii* only expresses the Tg*NTPase 1* and *3*, which is under the control of an active promoter solely present in those alleles [13, 44]. This is indicative of a similar promoter structure for the Nc*NTPase* alleles. However, our analysis indicates a low percentage of identity between the promoter sequences of the Nc*NTPase* and Tg*NTPase* genes (< 50 %; data not shown). In addition, we have predicted the presence of several up-stream *cis*-acting elements (TGAGACGC). These motifs have shown to be essential for the transcription initiation of certain proteins from the surface and dense granules of *T. gondii* and *S. neurona* [45–47], and thus, their distribution may be relevant to regulate the transcription process. Nevertheless, promoter mapping by 5' deletion analysis would be necessary to establish the role of these sequences in the transcription of Nc*NTPases*.

Moreover, we have identified up to four different Nc*NTPase* alleles in the Nc-Liv isolate. Previous studies reported on the presence of three tandemly repeated copies of the Tg*NTPase* gene in *T. gondii* [22], and Asai and colleagues then also detected up to three different *loci* and different alleles of the Nc*NTPase* in *N. caninum* [20]. However, the Nc*NTPase* 2 and 3 copies show a reading frame shift caused by a single-nucleotide insertion, and thus, it is unlikely that both genes are properly translated. Remarkably, it has been recently described that gene duplications are notoriously underestimated due to collapsing of the assembly in regions containing tandemly duplicated clusters of similar genes [48]. More specifically, Adomako-Ankomah and colleagues have evidenced the existence of expanded *loci* carrying a variable number of alleles coding for the TgNTPase protein, the numbers of which depend on the isolate. Similarly, several recognized virulent factors of *T. gondii* are clonally expanded and differ in copy number and sequences among isolates [48]. These findings support our evidence on the wide variety of transcribed Nc*NTPase* alleles that we have observed, which could be harboured within the Nc*NTPase 1 loci*. Consistent with this, we failed to amplify the whole Nc*NTPase 1* gene (coding sequence and flaking regions) by PCR, even when employing specific long-range PCR methods (data not shown). These findings suggest that the Nc*NTPase* allele repertoire may vary among *N. caninum* isolates, and if so, the availability of different genome annotations from diverse *N. caninum* isolates would be highly desirable. However, to date only the Nc-Liv genome has been fully sequenced [12].

In addition, we have demonstrated the presence of diverse protein species of the NcNTPase in the Nc-Liv isolate by 2-DE immunoblot. This is in accordance with previous proteomic studies in which the NcNTPase protein was detected in different protein spots [21, 49, 50]. Both the presence of multiple Nc*NTPase* alleles, and the detection of different protein species, suggest that *N. caninum* expresses different isoforms of the protein as reported earlier for *T. gondii* [51]. According to this, further studies should be carried out in order to clarify the impact of the Nc*NTPase* polymorphism on parasite pathogenicity.

By immunogold TEM, the NcNTPase was localized to the dense granules of intracellular tachyzoites employing a specific α-NcNTPase antibody. These findings are in accordance with a previous study in which a similar pattern was observed when extracellular tachyzoites were labelled with an α-TgNTPase antibody [20]. In addition, we performed a detailed immunofluorescent tracing of the NcNTPase and NcGRA7 proteins throughout the lytic cycle by using three different fixation protocols. NcNTPase and NcGRA7 secretion was easily detected during early invasion, PV maturation, and egress no matter which fixation was employed. Besides, both proteins associated with the vacuole periphery, indicating that they might interact with the PVM, as previously reported for TgNTPase [22] and the NcGRA7 proteins [10]. Moreover, as shown by the co-localisation studies, the temporal distribution of the NcNTPase and NcGRA7 proteins was distinct during invasion and egress, suggestive of differential protein trafficking at these stages of the lytic cycle. However, NcNTPase is clearly a canonical GRA protein according to earlier described criteria [9]: it has a relatively low molecular weight, the protein carries a N-terminal signal peptide, it colocalized with NcGRA7 within the dense granules, was secreted into the tachyzoite PV, and, similar to NcGRA7, remained there until parasites underwent egress. The possible existence of distinct secretory granules has been recently suggested for the ‘canonical’ GRA of dense granule and other “GRA” proteins located in uncharacterized cytoplasmic vesicles [9]. If NcNTPase tracking involves different dense granule subpopulations throughout the lytic cycle should be broadly investigated.

The expression of Nc*NTPase* and Nc*GRA7* transcripts was quantified in infected MARC-145 throughout the lytic cycle. For both, the highest mRNA levels were detected at 6 and 56 hpi, coinciding with egress and/or early invasion. Therefore, NcNTPase and NcGRA7 function may be necessary to guarantee the lytic cycle progression. A similar expression pattern was described previously for the rhoptry proteins NcROP40 and NcROP2Fam-1 [32], highlighting the relevance of rhoptry and dense granule proteins for the invasion and/or the immediately following processes in *N. caninum*. This is also in accordance with previous studies carried out with *Plasmodium falciparum* and *T. gondii*, that showed that the changes in mRNA levels at specific points of the lytic cycle have a strong influence on the parasite’s developmental transitions [52, 53]. In line with this, a modal switch from expression of proteins involved in invasion and motility of *T. gondii* has been also described in egressed tachyzoites [54, 55].

On the other hand, we quantified the NcNTPase and NcGRA7 mRNA levels after inducing *N. caninum* egress in vitro by DTT treatment [32, 56]. Significant increases in their mRNA levels were observed after DTT supplementation, suggesting that both proteins may be relevant during the egress or subsequent phases. Similar findings have been reported earlier for the NcROP2Fam-1 protein, whilst Nc*ROP40* mRNA expression was not affected by DTT supplementation [32]. Interestingly, *T. gondii* secretes the reducing agent glutaredoxin as replication increases. In fact, this compound is able to activate the TgNTPase in vitro. Similarly, DTT has been also shown to activate the TgNTPase protein in vitro and to trigger egress of tachyzoites [57, 58]. Although the processes governing egress are not fully understood, intracellular calcium levels appear to trigger the abrupt exit of parasites from the PV, which is accompanied by a rapid decrease in host cell ATP mediated by TgNTPase activation [59]. Hence, we hypothesize that egress regulation in *N. caninum* relies on NcNTPase activation, which is accompanied by an increase in the expression of secreted effectors (NcGRA7 and NcROP2Fam-1) necessary to accomplish new invasion waves in the neighbouring cells [32]. In fact, TgNTPase has been previously suggested to act as a timer for the *T. gondii* lytic cycle [42]. Since egress and invasion represent critical processes in which parasites are highly exposed to the host immune system, NcNTPase secretion could counteract the development of inflammatory responses to avoid tachyzoite clearance. Interestingly, it has been suggested that the TgNTPase may suppress the local host immune responses [37, 41–43], supporting this hypothesis.

In contrast to microneme proteins, whose secretion can be activated by calcium ionophores (A23187 and ethanol), *T. gondii* GRA proteins are constitutively released in a calcium-independent and a temperature-dependent mode [60–62]. Similarly, DTT exposure has recently been shown to specifically induce the secretion of the NcMIC2 protein [32]. This compound is an egress inducer that reduces the TgNTPase protein in vivo, and induces a rapid depletion of host cell ATP with a concurrent calcium flux [40, 57]. However, the effect of these treatments on Tg/NcNTPase secretion had not been tested so far. Accordingly, we found that NcNTPase and NcGRA7 secretion occurs in tachyzoites undergoing egress regardless of the applied treatment (A23187, ethanol, or DTT at 37 ° C). In addition, secretion was also found in tachyzoites maintained at 4 ° C, suggesting that the NcNTPase is constitutively secreted during the egress stage. Similar findings have been previously reported for the SnNTPase protein from *S. neurona*, which can be detected on secreted fractions without further treatment [38].

Intriguingly, both NcNTPase and NcGRA7 are phosphoproteins. This protein modification may indicate a common regulation mechanism necessary for their participation within the lytic cycle. We have recently employed the same Phos-Tag 1-DE approach to determine the phosphorylation state of the two rhoptry proteins NcROP40 and NcROP2Fam-1, and found that NcROP2Fam-1 protein was also phosphorylated [32]. Previous studies have also demonstrated the phosphorylation of TgROP2, TgROP4 and TgGRA7, but only in intracellular parasites [63–65]. Interestingly, phosphorylation of TgGRA6 has been shown to coincide with its association with the PVM [66, 67], and this could also be the case for NcNTPase and NcGRA7 proteins, which are also localized at the periphery of the PV. Nevertheless, TgGRA7 was shown to associate with the PVM independently of its phosphorylation status [63, 65, 68]. In fact, it was suggested that TgGRA7 phosphorylation might regulate the formation of complexes with other GRA proteins to facilitate secretion of transmembrane-domain containing proteins [69].

## Conclusions

In summary, we have unraveled, at least partially, the complex Nc*NTPase* genome organization and demonstrate that three different *loci* and allele varition of the Nc*NTPase* gene exist. However, the improvement of the current gene annotation within the Nc-Liv genome and the incorportation of new fully sequenced isolates would be highly desirable in order to clarify more accurately grade of gene expansion and polymorphism of the NcNTPase genes. The present work aimed to characterize the NcNTPase and NcGRA7 proteins through an integrative and descriptive approach in the context of the host-parasite relationship. NcNTPase and NcGRA7 are up-regulated, secreted and phosphorylated during egress and early invasion, which suggests that both are likely involved in these and the subsequent phases of the lytic cycle. Nevertheless, the specific role of the NcNTPase and NcGRA7 proteins remains to be elucidated. In this sense, reverse genetics would be useful to determine the NcNTPase and NcGRA7 function, and to assess the impact of NcNTPase expression in the pathogenicity displayed among isolates.

## Additional files

Additional file 1: Clustal alignment of the genome Nc*NTPase* and allele sequences from GeneBank database and this study. Primers for sequencing clones 1, 2, and 3 are displayed in the table below. (DOCX 46 kb) Additional file 2: Clustal alignment of the up-stream regions of the Nc*NTPase 1*, *2* and *3*. Cis-acting elements (yellow) and the ORF of the Nc*NTPase* genes (light blue) are displayed on the figure. Specific primers were designed to amplify the up-stream sequences of the Nc*NTPase 1*, *2* and *3* as indicated in the table below. Forward primers were specific for the Nc*NTPase 1*, *2* and *3* sequences, whilst reverse primer was common for all the copies. PCR amplification yielded a single fragment with the expected molecular weight (see figure below). (DOCX 53 kb)
