# Supplementary material for: The tandemly repeated NTPase (NTPDase) from Neospora caninum is a canonical dense granule protein whose RNA expression, protein secretion and phosphorylation coincides with the tachyzoite egress
Source: Parasit Vectors. 2016 Jun 21;9:352. doi: 10.1186/s13071-016-1620-4 (PMC4915099; doi:10.1186/s13071-016-1620-4)
Supplement: Additional file 1: — Clustal alignment of the genome NcNTPase and allele sequences from GeneBank database and this study. Primers for sequencing clones 1, 2, and 3 are displayed in the table below. (DOCX 46 kb) [file 13071_2016_1620_MOESM1_ESM.docx]

**10 20 30 40 50 60 70 80 90 100 110 120**

**....|....|....|....|....|....|....|....|....|....|....|....|....|....|....|....|....|....|....|....|....|....|....|....|**

**>NTP1_chrXII**  **ATGGGGG-TTCCTATCTGGGCTGGTTGTTTGCTGGTGTTGGGTGTGAGTTTGATCCTCCCTTTAGGAGGCTGTGCCGACGAGCCAGCGACACTTCGCGGAGTGAGCGCCGAGACAGAAAA**

**>NTP2_chrXII**  **.......G............................................................A.......................................G...........**

**>NTP3_chrXII**  **.......G...T............................................................................................................**

**>XM_003886392.1** **.......-................................................................................................................**

**>AB525222.1**  **-------------------------------------------------------------------------...................................G...........**

**>AB010444.1**  **-------------------------------------------------------------------------...................................G...........**

**>Clone 1**  **-------------------------------------------------------------------------...............................................**

**>Clone 2**  **-------------------------------------------------------------------------...............................................**

**>Clone 3**  **-------------------------------------------------------------------------...............................................**

**130 140 150 160 170 180 190 200 210 220 230 240**

**....|....|....|....|....|....|....|....|....|....|....|....|....|....|....|....|....|....|....|....|....|....|....|....|**

**>NTP1_chrXII**  **ACATATTTCTGCCGGTAAAGCACGCCTGCAGGACCTCCGCGATGCGGAACGTCGCTGTCATGATGCCTGGCAAGCAATTGTTGTGATCGATGGAGGCAGTAGTGCGACGCGGACGAATGT**

**>NTP2_chrXII**  **........................................................................................................................**

**>NTP3_chrXII**  **........................................................................................................................**

**>XM_003886392.1** **........................................................................................................................**

**>AB525222.1**  **........................................................................................................................**

**>AB010444.1**  **...................................................................................................................G....**

**>Clone 1**  **........................................................................................................................**

**>Clone 2**  **........................................................................................................................**

**>Clone 3**  **........................................................................................................................**

**250 260 270 280 290 300 310 320 330 340 350 360**

**....|....|....|....|....|....|....|....|....|....|....|....|....|....|....|....|....|....|....|....|....|....|....|....|**

**>NTP1_chrXII**  **ATTTCTGGCGAAGACCCGGTCATGCCCACGTGGAGGCCGACACATTGACCCCGACAGTATTCGACTCCTCGGCGCGGGTAAGCGCTTCGCAGGACTTCGTGGAGTTTTAGAAAGCTGGCT**

**>NTP2_chrXII**  **........................................................................................................................**

**>NTP3_chrXII**  **........................................................................................................................**

**>XM_003886392.1** **........................................................................................................................**

**>AB525222.1**  **........................................................................................................................**

**>AB010444.1**  **........................................................................................................................**

**>Clone 1**  **........................................................................................................................**

**>Clone 2**  **........................................................................................................................**

**>Clone 3**  **........................................................................................................................**

**370 380 390 400 410 420 430 440 450 460 470 480**

**....|....|....|....|....|....|....|....|....|....|....|....|....|....|....|....|....|....|....|....|....|....|....|....|**

**>NTP1_chrXII**  **AGACGCGTACGCTGGAGAGGACTGGGAGTCGAGGTCTGTTGATTCCAAGCGTCTCTTCCAGCACGTTCCTGAGATGGAAGACAGTGCAAGGGGCCTCATGCAGCTCTTGGAGGACGACGC**

**>NTP2_chrXII**  **........................................................................................................................**

**>NTP3_chrXII**  **........................................................................................................................**

**>XM_003886392.1** **........................................................................................................................**

**>AB525222.1**  **........................................................................................................................**

**>AB010444.1**  **........................................................................................................................**

**>Clone 1**  **........................................................................................................................**

**>Clone 2**  **........................................................................................................................**

**>Clone 3**  **........................................................................................................................**

**490 500 510 520 530 540 550 560 570 580 590 600**

**....|....|....|....|....|....|....|....|....|....|....|....|....|....|....|....|....|....|....|....|....|....|....|....|**

**>NTP1_chrXII**  **AGTTCGCATCTTGGACGAGAAACTCACTGAGGAACAAAAGGTTCAGGTTCAAGCGATGGGCGTTCCAGTCCTGCTGTGCAGCACGGCAGGAGTTCGTGATTTCCACGACTGGTACCGCGA**

**>NTP2_chrXII**  **........................................................................................................................**

**>NTP3_chrXII**  **........................................................................................................................**

**>XM_003886392.1** **........................................................................................................................**

**>AB525222.1**  **........................................................................................................................**

**>AB010444.1**  **........................................................................................................................**

**>Clone 1**  **........................................................................................................................**

**>Clone 2**  **........................................................................................................................**

**>Clone 3**  **........................................................................................................................**

**610 620 630 640 650 660 670 680 690 700 710 720**

**....|....|....|....|....|....|....|....|....|....|....|....|....|....|....|....|....|....|....|....|....|....|....|....|**

**>NTP1_chrXII**  **AGCCCTCTTTGTCATTCTTCGCTTTCTCATCAATCACCCGAAGCCTGGCCACGGGTACAAATTCTTCACCAACCCCGAATGGACCCGACCGATCACGGGCGCTGAGGAAGGTCTGTACGC**

**>NTP2_chrXII**  **........................................................................................................................**

**>NTP3_chrXII**  **........................................................................................................................**

**>XM_003886392.1** **........................................................................................................................**

**>AB525222.1**  **........................................................................................................................**

**>AB010444.1**  **........................................................................................................................**

**>Clone 1**  **........................................................................................................................**

**>Clone 2**  **........................................................................................................................**

**>Clone 3**  **........................................................................................................................**

**730 740 750 760 770 780 790 800 810 820 830 840**

**....|....|....|....|....|....|....|....|....|....|....|....|....|....|....|....|....|....|....|....|....|....|....|....|**

**>NTP1_chrXII**  **ATTTCTCGCGCTCAACCATCTTTCGGGGCGGTTAGGCGAAGACCCAGCTAGGTGTTACGTTGATGAATACGGGATGAAGCAGTGCCGCAATGACCTTGTTGGCGTGGTTGAAGTGGGCGG**

**>NTP2_chrXII**  **........................................................................................................................**

**>NTP3_chrXII**  **........................................................................................................................**

**>XM_003886392.1** **........................................................................................................................**

**>AB525222.1**  **........................................................................................................................**

**>AB010444.1**  **........................................................................................................................**

**>Clone 1**  **........................................................................................................................**

**>Clone 2**  **........................................................................................................................**

**>Clone 3**  **........................................................................................................................**

**850 860 870 880 890 900 910 920 930 940 950 960**

**....|....|....|....|....|....|....|....|....|....|....|....|....|....|....|....|....|....|....|....|....|....|....|....|**

**>NTP1_chrXII**  **TGCTTCTACCCAAATCGTTTTTCCACTACAGGACGGCACTGCCCTGCCCTCGTCCATCCGTGCCGTTAACCTGCAGCACGAACGCTTTCTCCCATCGCGTTTTCCGAGTGCCGACGTCAT**

**>NTP2_chrXII**  **........................................................................................................................**

**>NTP3_chrXII**  **..........................................................................................................T.............**

**>XM_003886392.1** **........................................................................................................................**

**>AB525222.1**  **........................................................................................................................**

**>AB010444.1**  **..........................................................................................................T.............**

**>Clone 1**  **........................................................................................................................**

**>Clone 2**  **........................................................................................................................**

**>Clone 3**  **..........................................................................................................T.............**

**970 980 990 1000 1010 1020 1030 1040 1050 1060 1070 1080**

**....|....|....|....|....|....|....|....|....|....|....|....|....|....|....|....|....|....|....|....|....|....|....|....|**

**>NTP1_chrXII**  **ATCGGTTTCTTTTATGCAGCTGGGTGTGGCCAGTTCCTCCGGACTGTTTTTCAAGGAGCTGTGCAGTAACGCCGAATTTCGGCATCAGGGGATTTGCTATAATCCCTGTATTTTCAGAGG**

**>NTP2_chrXII**  **........................................................................................................................**

**>NTP3_chrXII**  **........................................................................................................................**

**>XM_003886392.1** **........................................................................................................................**

**>AB525222.1**  **........................................................................................................................**

**>AB010444.1**  **........................................................................................................................**

**>Clone 1**  **........................................................................................................................**

**>Clone 2**  **........................................................................................................................**

**>Clone 3**  **........................................................................................................................**

**1090 1100 1110 1120 1130 1140 1150 1160 1170 1180 1190 1200**

**....|....|....|....|....|....|....|....|....|....|....|....|....|....|....|....|....|....|....|....|....|....|....|....|**

**>NTP1_chrXII**  **CTTCCGACAGGCCTGTTCCGCGGGCGATGTCGAGATCCTTCCAGACGGAACCATAGTTGTGGATGAAGATGTGCGAAAGAACAAGCTGAAGCCGGTGGCGACATCTTGCTCAGCGAACAA**

**>NTP2_chrXII**  **........................................................................................................A...............**

**>NTP3_chrXII**  **........................................................................................................................**

**>XM_003886392.1** **........................................................................................................................**

**>AB525222.1**  **........................................................................................................A...............**

**>AB010444.1**  **........................................................................................................................**

**>Clone 1**  **........................................................................................................A...............**

**>Clone 2**  **........................................................................................................A...............**

**>Clone 3**  **........................................................................................................A...............**

**1210 1220 1230 1240 1250 1260 1270 1280 1290 1300 1310 1320**

**....|....|....|....|....|....|....|....|....|....|....|....|....|....|....|....|....|....|....|....|....|....|....|....|**

**>NTP1_chrXII**  **TCCGGAAATTAGTTTCAAGGCAATGAATGAAATGCAATGTCGCGAAAACAAGATTGATCCGACGAAATCACTCGCGGAGCGGCTGAGGATCGACGACTGCTTCCAGATTGTAGGAACTGG**

**>NTP2_chrXII**  **.................................T..........T...........................................................................**

**>NTP3_chrXII**  **........................................................................................................................**

**>XM_003886392.1** **........................................................................................................................**

**>AB525222.1**  **.................................T..........T...........................................................................**

**>AB010444.1**  **........................................................................................................................**

**>Clone 1**  **.................................T..........T...........................................................................**

**>Clone 2**  **.................................T..........T...........................................................................**

**>Clone 3**  **.................................T..........T...........................................................................**

**1330 1340 1350 1360 1370 1380 1390 1400 1410 1420 1430 1440**

**....|....|....|....|....|....|....|....|....|....|....|....|....|....|....|....|....|....|....|....|....|....|....|....|**

**>NTP1_chrXII**  **TGATTTCGACACATGCCAAGCCCAGGTAGAAGAACTTCTCGTCAGTCCCAGGTTTCCACTTCCAGCGAACATTGAGGCAGCGTCGTCAGGCTTCGAATCTGTTGGTCAGGTTTTCAAGTT**

**>NTP2_chrXII**  **........................................................................................................................**

**>NTP3_chrXII**  **........................................................................................................................**

**>XM_003886392.1** **........................................................................................................................**

**>AB525222.1**  **........................................................................................................................**

**>AB010444.1**  **........................................................................................................................**

**>Clone 1**  **........................................................................................................................**

**>Clone 2**  **........................................................................................................................**

**>Clone 3**  **........................................................................................................................**

**1450 1460 1470 1480 1490 1500 1510 1520 1530 1540 1550 1560**

**....|....|....|....|....|....|....|....|....|....|....|....|....|....|....|....|....|....|....|....|....|....|....|....|**

**>NTP1_chrXII**  **CGCGTCTACGGCGTCGCCGATGGTCATTACAGGAGGAGCGATGTATGCGAGCATCAGCACAATGCAAGGTTTCGGACTTCTTCCGAAAGACTTCCAGGGTGATCTAGAACAACTGATAGC**

**>NTP2_chrXII**  **......................................................................C.................................................**

**>NTP3_chrXII**  **......................................................................C........................C........................**

**>XM_003886392.1** **........................................................................................................................**

**>AB525222.1**  **......................................................................C.................................................**

**>AB010444.1**  **......................................................................C........................C........................**

**>Clone 1**  **......................................................................C.................................................**

**>Clone 2**  **......................................................................C.................................................**

**>Clone 3**  **......................................................................C.................................................**

**1570 1580 1590 1600 1610 1620 1630 1640 1650 1660 1670 1680**

**....|....|....|....|....|....|....|....|....|....|....|....|....|....|....|....|....|....|....|....|....|....|....|....|**

**>NTP1_chrXII**  **TGCATCACGTACATACTGTTCGTCTCCAGTAGTTAACAGTGGAGATGGCCTTGTCATTCAGTTGCCAAACGCGGAACAAAAGCTGACTAGTATGAACTACGACTTATGCAAGACGATCGC**

**>NTP2_chrXII**  **......................................A..................................................................G..............**

**>NTP3_chrXII**  **.........................................................................................................G..............**

**>XM_003886392.1** **........................................................................................................................**

**>AB525222.1**  **......................................A..................................................................G..............**

**>AB010444.1**  **.........................................................................................................G..............**

**>Clone 1**  **.........................................................................................................G..............**

**>Clone 2**  **......................................A..................................................................G..............**

**>Clone 3**  **......................................A..................................................................G..............**

**1690 1700 1710 1720 1730 1740 1750 1760 1770 1780 1790 1800**

**....|....|....|....|....|....|....|....|....|....|....|....|....|....|....|....|....|....|....|....|....|....|....|....|**

**>NTP1_chrXII**  **GCTGACTGTTTCGCTGCTTCAGCACATGGAGGCAGGCGAGCATAAACCGTCATCCATTTCCTGGCAAAAAACCGTTGTGGGACCAGATGGAAAACCACGTGCTGATCTTGGGTGGCATGT**

**>NTP2_chrXII**  **.....................................................................................................................C..**

**>NTP3_chrXII**  **................A......................................................G................................................**

**>XM_003886392.1** **........................................................................................................................**

**>AB525222.1**  **.....................................................................................................................C..**

**>AB010444.1**  **................A......................................................G................................................**

**>Clone 1**  **................A......................................................G................................................**

**>Clone 2**  **.....................................................................................................................C..**

**>Clone 3**  **.....................................................................................................................C..**

**1810 1820 1830 1840 1850 1860 1870 1880**

**....|....|....|....|....|....|....|....|....|....|....|....|....|....|....|....|..**

**>NTP1_chrXII**  **TGGGGCAATTCTTCATCGTGTCCTCTTCACGGAAGAATGGGGACGTACAGCTTATGAGACGGGATTTACCTACAACATGTGA**

**>NTP2_chrXII**  **..................................................................................**

**>NTP3_chrXII**  **..................................................................................**

**>XM_003886392.1** **.....................................................................A............**

**>AB525222.1**  **..................................................................................**

**>AB010444.1**  **..................................................................................**

**>Clone 1**  **..................................................................................**

**>Clone 2**  **..................................................................................**

**>Clone 3**  **..................................................................................**

| **Primer** | **Sequence** |
| --- | --- |
| Fw-T7 | TAATACGACTCACTATAGGG |
| Fw-1 | GAGCTCATGGCCGACGAGCCAGCGACACTT |
| Fw-int-1 | CGTCGTCAGGCTTCGAATCTGTT |
| Fw-int-2 | GATTTCCACGACTGGTACCG |
| Rv-SP6 | ATTTAGGTGACACTATAG |
| Rv-1 | GGCCTTAATTAATCACATGTTGTAGGTAAATCCCG |
| Rv-int-1 | TTCTTCTACCTGGGCTTGGC |
| Rv-int-2 | ACGCTTGGAATCAACAGACCT |
