# Supplementary material for: The tandemly repeated NTPase (NTPDase) from Neospora caninum is a canonical dense granule protein whose RNA expression, protein secretion and phosphorylation coincides with the tachyzoite egress
Source: Parasit Vectors. 2016 Jun 21;9:352. doi: 10.1186/s13071-016-1620-4 (PMC4915099; doi:10.1186/s13071-016-1620-4)

**pre-NcNTP1**  **ATGACTACCGACACCAAGTTCCTTATGATTGCTGTACAAATACAACCAGCTATAGATTAAAGAATCTACACGCGAGTGTAACGAAAAAACTGGAGCCTAT**

**pre-NcNTP2**  **---------------------------------------------------------------------------------CGAAAA-------------**

**pre-NcNTP3**  **----------------------------------------------------------------------CGCGAGTGTAACGAAAAAACTGGAGCCTAT**

**Consensus**   **********

**pre-NcNTP1**  **TTCCCAGCGGAGAAGCGCAATCTTTTGCCGTGCCACACACAGTCAGCAAGGTGAGGGACTGTGCCCGTGCAGCCCATCCTACTCTATACCAGAAACAGGT**

**pre-NcNTP2**  **--CGCGGTGTAGAGGC---ATCCTC--------------------------------------CTCGCGGGGCCCATTCTA--------CAGAAGCAAA-**

**pre-NcNTP3**  **TTCCCAGCGGAGAAGCGCAATCTTTTGCCGTGCCACACACAGTCAGCAAGGTGAGGGACTGTGCCCGTGCAGCCCATCCTACTCTATACCAGAAACAGGT**

**Consensus**   *** * * * *** ** *** * * ** * ****** *** ***** ****

**pre-NcNTP1**  **ACTGCGAGCGACACTGTGCGAAATTGTGTTTGTGTTTTGGGGGAAAAAACTTAAGACTAGCTAACGCTTCACGTCGCAGCACCGCCTCTGTAGATCATCC**

**pre-NcNTP2**  **----------------------------------------------------AGGGCGAATCAACGGTGCACGCC-------------------------**

**pre-NcNTP3**  **ACTGCGAGCGACACTGTGCGAAATTGTGTTTGTGTTTTGGGGGAAAAAACTTAAGACTAGCTAACGCTTCACGTCGCAGCACCGCCTCTGTAGATCATCC**

**Consensus**   *** * * * **** * **** ***

**pre-NcNTP1**  **ACAAACAGATCCTCTACACATCGCTTCCCCGACCCAGCCTCGACAATCTACAAGCAACCGCACTGTGCGGCAGCCGTGACGTGCTCACTGAGTCACTTCA**

**pre-NcNTP2**  **----------------------------------------------------------------------------------------------------**

**pre-NcNTP3**  **GCAAACAGATCCTCTACACATGGCTTCCCCGACCCAGCCTCGACAATCTACAAGCAACCGCACTGTGCGGCAGCCGTGACGTGCTCACTGAGGCACTTCA**

**Consensus**

**pre-NcNTP1**  **CAAACTCCGGACGATAGACGCAACCCCCTCTTATACTAAAGGATCATGGTCATCTTATTACCTTTCTTCAATGACGACTTCCCTCCATTCCCCGACCCAC**

**pre-NcNTP2**  **----------------------------------------------------------------------------------------------------**

**pre-NcNTP3**  **CAAACTCCGGACGATAGACGCAACCCCCTCTTATACTAAAGGATCATGGTCATCTTATTACCTTTCTTCAATGACGACTTCCCTCCATTCCCCGACCCAC**

**Consensus**

**pre-NcNTP1**  **CGCACTCCGATATCCTCGATTGTGGAACACCGCGATGTTTGTGACTGAGACCCCTAATTCGCTGACGCCTGGCTCCTCCTCCATTGTCTATGGCAGTACA**

**pre-NcNTP2**  **----------------------------------------------------------------------------------------------------**

**pre-NcNTP3**  **CGCACTCCGATATCCTCGGTTGTGGAACACCGCGATGTTTGTGACTGAGACCCCTAATTCGCTGACGCCTAGCTCCTCCTCCATTGTCTATGGCAGTACA**

**Consensus**

**pre-NcNTP1**  **ACAGTTTCCTCCTAATCGGTTAAGTCTATTCTATCCCACGTTTGTGTTCTTTAGTTTGAAATGACGCCGGTTTCGGTTCTGCAAGTTTGTAGTGTCGTCG**

**pre-NcNTP2**  **---------------------------------------------------------------ACGCCGGC-----------------------------**

**pre-NcNTP3**  **ACAGTTTCCTCCTAATCGGTTAAGTCTATTCTATCCCACGTTTGTGTTCTTTAGTTTGAAATGACGCCGGTTTCGGTTCTGCAAGTTTGTAGTGTCGTCG**

**Consensus**   ***********

**pre-NcNTP1**  **TTGCAGCACAAGGCATGTGATCTACTCGGCTGTAGTGCCCCGCCATGCGAACCGCTCGTTCACACTTGGTAGCATACTATCAACGGCAGAACACGAGAGG**

**pre-NcNTP2**  **----------------------------------------------------------------------------------------------------**

**pre-NcNTP3**  **TTGCAGCACGAGGCATGTGATCTACTCGGCTGTAGTGCCCCGCCATGCGAACCGCTCGTTCACACTTGGTAGCATACTATCAACGGCAGAACACGAGAGG**

**Consensus**

**pre-NcNTP1**  **AAAACCAATTATTTCCCCATTCACCATCATTCGCCATCACAGCGCTCCGTGTCGTAGCGTGGAGACGATGGCGGCACAAGGCATCCTCAGAACTTTTGTC**

**pre-NcNTP2**  **-------------------------------------------------------------------------------GGCATCCTCAGAACTTTTGTA**

**pre-NcNTP3**  **AAAACCAATTATTTCCCCATTCACCATCATTCGCCATCACAGCGCTCCGTGTCGTAGCGTGGAGACGATGGCGGCACAAGGCATCCTCAGAACTTTTGTC**

**Consensus**   ************************

**cis**

**pre-NcNTP1**  **GTTCTGTTGCATGGGCGTCAACCTCTGCCTATTCTAGCGCGGGACCACAGTTGCGGGTTCTACGGGGACCCGGCGGCCGAACGTTCACCACGGCAATTGA**

**pre-NcNTP2**  **GTTCTGTTGCATGGGCATCAGCCTTTGCCTATTCTAGCGCGTGGCCACAGTTGCGGGTTCTACGGGGACCCGGCGGCCGAACGTTCACCACGGCAATTGA**

**pre-NcNTP3**  **GTTCTGTTGCATGGGCGTCAACCTCTGCCTATTCTAGCGCGGGACCACTGTTGCGGGTTCTACGGGGACCCGGCGGCCGAACGTTCACCACGGCAATTGA**

**Consensus**  ****************** *** *** **************** * **** *****************************************************

**-elem** **cis-elem** **cis-elem** **cis-elem**

**pre-NcNTP1**  **GACGCACGCATCGTTTATGCGACACCAGCACTCTCTG------------------------CCGATTGAGACGCATTGCTGAACTCCCAATGAGACGCGT**

**pre-NcNTP2**  **GACGCACACATCGTTCATGCGACACTAGCACTCTCTG-------------------GGAAGCCTATTGAGACGCATTGCTGAACCCTCATTGAGACGCGT**

**pre-NcNTP3**  **GACGCACGCATCGTTTATGCGACACCAGCACTCTCTGCCGATTGAGACGCATTACTGAACTCCCAATGAGACGCATTGCTGAACCCTCATTGAGACGCGT**

**Consensus**  ********* ******* ********* *********** ** * ****************** * ** ************

**pre-NcNTP1**  **CCAGAACGAACCCGATCCCGTTTCCGGGGCCCAGCGACGACATTCGGGGCACCGTTAACGTCGCCCAGCGCCTCGTGTGAGACTGCCACTTGTTCCTGCT**

**pre-NcNTP2**  **CCAGAACAAA------CCCGTTTCCGGGGCCCAGCGACGACATTCGGGGCACCGTTAACGTCGCCCAGCGCCTCGTGTGAGACTGCCACGTGTTCCTGCT**

**pre-NcNTP3**  **CCAGAACGAACCCGATCCCGTTTCCGGGGCCCAGCGGCGACATTCGGGGCACCGTTAACGTCGCCCAGCCCCTCGTGTGAGACTGCCACGTGTTCCTGCT**

**Consensus**  ********* ** ******************** ******************************** ******************* ************

**pre-NcNTP1**  **GTTCCTGCACGCCCTGTGGTCTATTCCTCATTGTCTCGGTTCCCGGTAGAACTAGGTTCGAGTCCCAGTTTTTTGAGAGTTACTAGAGAGTTGCCTGAGT**

**pre-NcNTP2**  **GTTCCTGCACGCCCTGTGGTCTATTCCTCATTGTCTCGGTTCCCGGTAGAACTAGGTTCGAGTCCCAGTTTTTTGAGAGTTACTAGAGAGTTGCCTGAGT**

**pre-NcNTP3**  **GTTCCTGCACGCCCTGTGGTCTATTCCTCATTGTCTCGGTTCCCGGTAGAACTAGGTTCGAGTCCCAGTTTTTTGAGAGTTACTAGAGAGTTGCCTGAGT**

**Consensus**  ********************************************************************************************************

**NTPase ORF**

**pre-NcNTP1**  **TACCTTGTCGTTCTGCATCTTCCTGTCTGTTGCCCGTATTCAAGTAGCCACGAAAAT-GGGGGTTCCTATCTGGGCTGGTTGTTTGCTGGTGTTGGGTGT**

**pre-NcNTP2**  **TACCTTGTCGTTCTGCATCTTCCTGTCTGTTGCCCGTATTCAAGAAGCCACGAAAATGGGGGGTTCCTATCTGGGCTGGTTGTTTGCTGGTGTTGGGTGT**

**pre-NcNTP3**  **TACCTTGTCGTTCTGCATCTTCCTGTCTGTTGCCCGTATTCAAGAAGCCACGAAAATGGGGGGTTCTTATCTGGGCTGGTTGTTTGCTGGTGTTGGGTGT**

**Consensus**  ********************************************** ************ ******** ***********************************

| Nc*NTPase*  copy | Forward primer | Reverse primer | Expected amplicon  length |
| --- | --- | --- | --- |
| 1 | CCTGCTCCATAAAGAACGTACCTA | CAATTGTTGTGATCGATGGAGG | 1599 bp |
| 2 | CGAAAACGCGGTGTAGAGG |  | 752 bp |
| 3 | GTCTCATAAATTTTGCAACCAGCGA |  | 1544 bp |


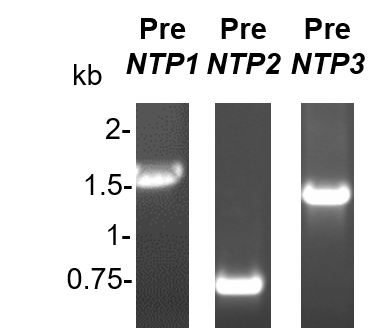

Supplement: Additional file 2: — Clustal alignment of the up-stream regions of the NcNTPase 1, 2 and 3. Cis-acting elements (yellow) and the ORF of the NcNTPase genes (light blue) are displayed on the figure. Specific primers were designed to amplify the up-stream sequences of the NcNTPase 1, 2 and 3 as indicated in the table below. Forward primers were specific for the NcNTPase 1, 2 and 3 sequences, whilst reverse primer was common for all the copies. PCR amplification yielded a single fragment with the expected molecular weight (see figure below). (DOCX 53 kb) [file 13071_2016_1620_MOESM2_ESM.docx]
